# Experimental Demonstration of Nanoscale Pillar Phononic Crystal-Based Reflector for Surface Acoustic Wave Devices

**DOI:** 10.3390/mi16060663

**Published:** 2025-05-31

**Authors:** Temesgen Bailie Workie, Lingqin Zhang, Junyao Shen, Jianli Jiang, Wenfeng Yao, Quhuan Shen, Jingfu Bao, Ken-ya Hashimoto

**Affiliations:** 1 School of Integrated Circuit Science and Engineering, University of Electronic Science and Technology of China, Chengdu 611731, China; 2 Tiantong Ruihong Technology Co., Ltd., No. 306, Haining 314499, China

**Keywords:** acoustic bandgap, phononic crystal, quality factor, reflector, resonator, RF filter, surface acoustic wave device

## Abstract

This article presents an investigation into the use of nanoscale phononic crystals (PnCs) as reflectors for surface acoustic wave (SAW) resonators, with a focus on pillar-based PnCs. Finite element analysis was employed to simulate the phononic dispersion characteristics and to study the effects of the pillar shape, material and geometric dimensions on achievable acoustic bandgap. To validate our concept, we fabricated SAW resonators and filters incorporating the proposed pillar-based PnC reflectors. The PnC-based reflector shows promising performance, even with smaller number of PnC arrays. In this regard, with a PnC array reflector consisting of 20 lattice periods, the SAW resonator exhibits a maximum bode-Q of about 1600, which can be considered to be a reasonably high value for SAW resonators on bulk 42° Y-X lithium tantalate (42° Y-X LiTaO_3_) substrate. Furthermore, we implemented SAW filters using pillar-based PnC reflectors, resulting in a minimum insertion loss of less than 3 dB and out-of-band attenuation exceeding 35 dB. The authors believe that there is still a long way to go in making it fit for mass production, especially due to issues related with the accuracy of fabrication. But, upon its successful implementation, this approach of using PnCs as SAW reflectors could lead to reducing the foot-print of SAW devices, particularly for SAW-based sensors and filters.

## 1. Introduction

Surface acoustic wave (SAW) devices have long been the benchmark for radio-frequency (RF) filtering applications, particularly in mobile communication devices such as cell phones. With the increasing demand for higher RF standards, there is a pressing need to enhance the performance of these devices. One of the key challenges in this endeavor is the reduction in the device foot-print while maintaining or improving performance. Metal reflective gratings are integral components of SAW devices and play pivotal role in determining the resonator’s performance, including the quality factor (Bode-Q) and insertion loss (IL) [1,2,3,4]. The standard way of achieving superior performance requires the use of a large number of metallic reflectors, which impacts the device’s size [1,2,4]. Significant efforts have been directed towards refining the design of acoustic reflectors, thereby reducing the size of SAW devices without affecting the performance [2,4].

In this context, phononic crystal (PnC)-based reflectors have emerged as an alternative solution due to their unique property of achieving elastic/acoustic bandgaps. The acoustic bandgap phenomenon allows for the inhibition of acoustic wave propagation in specific directions [5,6,7,8,9,10,11,12,13,14,15,16,17,18,19,20,21,22,23,24,25,26,27,28,29,30,31,32,33,34,35,36]. In this regard, extensive research has demonstrated the potential of PnCs in controlling the propagation of SAW, making PnCs particularly instrumental as reflectors for SAW devices [14,20,24,27,29,34,35,36].

Far too many studies have been conducted by the research community in the field to improve the theoretical understanding and demonstration of novel PnC structures that can achieve wider bandgaps with better reflection. However, the practical implementation of PnCs in SAW devices especially at higher frequencies remained limited, highlighting the need for further exploration and development.

This article presents a theoretical and experimental demonstration of SAW devices that employ PnC-based reflectors. Resonators and filters with nanoscale PnC-based reflectors, namely octagonal pillar (Op-PnC), cylindrical pillar (Cp-PnC), and square pillar (Sp-PnC) structures on a 42° Y-X-LiTaO^3^ (LT) substrate, were designed and fabricated. The findings indicate a promising future for PnCs as reflectors.

## 2. Device Modeling and Method of Calculation

### 2.1. Dispersion and Transmission Characteristics

**Figure 1:**
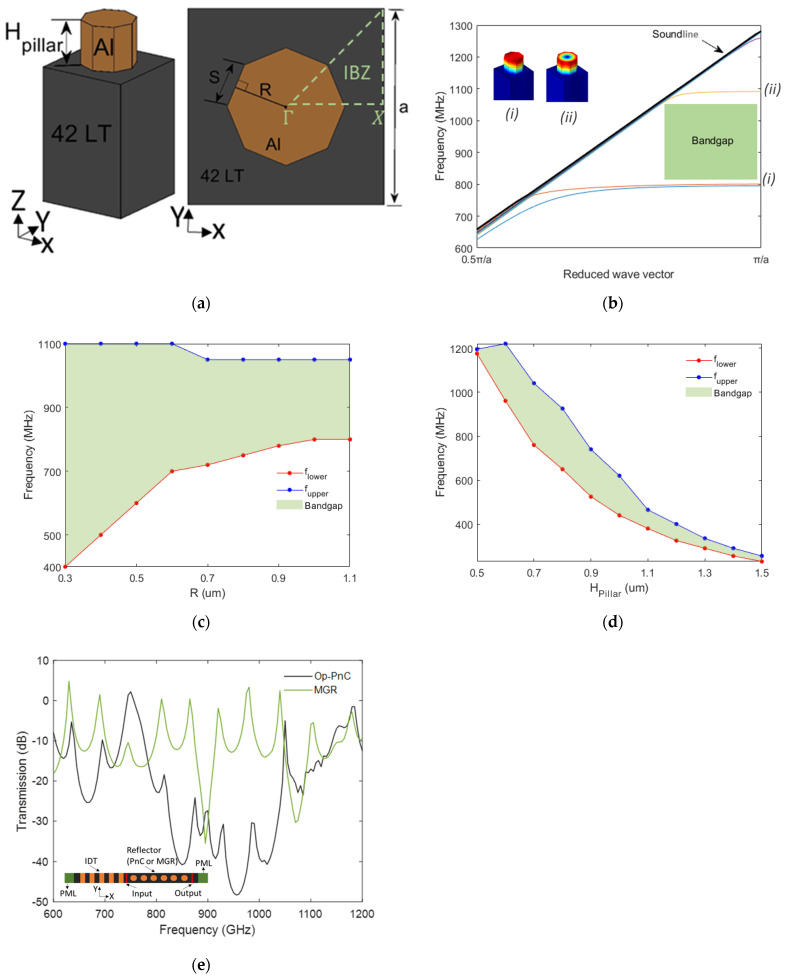
(**a**) Schematic representation of a unit cell Op−PnC. (**b**) Dispersion characteristics of Op−PnC with *H*_pillar_ = 700 nm and *ff* = 0.3. (**c**) Bandgap characteristics with radius (*R*) with constant *H*_pillar_ = 700 nm. (**d**) Bandgap characteristics with thickness *H*_pillar_ increases with constant *R* = 400 nm. (**e**) Transmission vs. frequency for SAWs through 10 Op−PnC lattice periods compared to MGR with equivalent length of reflector (inset schematic shows structure used for analysis).

The phononic dispersion characteristics of an octagonal pillar phononic crystal (Op-PnC) was investigated through finite element method (FEM) simulations (see refs [37,38] for a complete description of basic theories and PnC dispersion calculation mechanism). The PnC was constructed with 42° Y-X lithium tantalate (42° Y-X LiTaO_3_) as the substrate and aluminum (Al) for the pillar-based PnC. A schematic representation of the Op-PnC is shown in Figure 1a. The phononic dispersions were calculated using an Eigen frequency domain solver for the reduced wavevectors (*k*_x_ = *k* · *a*/π, where *k* is the wave vector and *a* is the lattice constant of the PnC) ranging from 0.5π/*a* to π/*a* across the Γ-X in the first Irreducible Brillouin Zone (IBZ) of the PnC square array, as shown in Figure 1b. The simulation involved an octagonal pillar PnC with a lattice constant (*a*) of 1300 nm, a height (*H*_pillar_) of 700 nm, and a radius (*R*) of 400 nm. These specific dimensions were chosen to optimize the bandgap characteristics of the Op-PnC, which is herein discussed in details. The plot in Figure 1b clearly shows a partial bandgap, shaded in green, between the lower bound frequency 800 MHz (*f*_lower_) and upper bound frequency 1091 MHz (*f*_upper_). The insets in Figure 1b present surface plots of total displacements for two SAW modes that support local resonances. These modes are marked as (*i*) and (*ii*) on the dispersion, corresponding to the frequencies that define the limits of the partial bandgap. The vibration modes at the lower bandgap limit show clear thickness dependence, while at the upper bandgap limit, they show both depth and radial dependence, suggesting that bandgap characteristics can be tuned using these two parameters. Figure 1c,d illustrate this adjustability by showing the frequency range of the lowest bandgap for varying the radii (*R*) and depth of the octagonal pillar (*H*_pillar_), respectively. In this regard, the filling fraction (*ff*), which is the ratio of the pillar volume to the total volume of the unit cell, plays a crucial role in determining the size and frequency of the acoustic bandgap. This is because the acoustic impedance (*Z*) changes with the change in the filling fraction, which further creates an acoustic impedance mismatch with the surrounding material, air in this particular case. In this regard, when the filling fraction is very low, the acoustic impedance is dominated by air and vice versa. As the radius (*R*) increases with a constant *H*_pillar_ = 700 nm, the bandgap width decreases. In this case, the analysis considers a minimum radius (*R*) of 300 nm, considering the current fabrication capability.

Conversely, as thickness *H*_pillar_ increases with constant *R* = 400 nm, the center frequency of the bandgap decreases, and the bandgap width initially increases, before starting to decrease, with optimum *H*_pillar_ = 700 nm to 900 nm, clearly showing a parabolic relation with *H*_pillar_. This can be attributed to the change in the mechanical properties (such as mass and stiffness) of the PnC with changes in the thickness of the pillar, which influences the propagation and reflection of acoustic waves. The minimum thickness considered is *H*_pillar_ = 500 nm, due to lack of acoustic bandgap below this value with the specific setup described above.

To assess the acoustic wave attenuation of the proposed PnC, we conducted simulations of SAW transmission through the Op-PnC, using a finite number of PnCs in the X-direction. Figure 1e shows the transmission for an Op-PnC comprising 10 lattice periods and a standard metal grating reflector (MGR) of equivalent reflector length. The inset of Figure 1e depicts a schematic representation of the structure utilized in these simulations. An interdigital transducer (IDT) was employed to excite source SAWs. The reflector is placed in the center, with the left and right sides set as input and output lines, respectively. The boundaries along both sides in the x-direction and the bottom in the z-direction were set as a perfectly matched layer (PML).

The transmission magnitude (in dB) was determined by calculating the ratio of the total acoustic wave intensity (i.e., total displacement) at the output line relative to that at the input line. Comparing the transmission characteristics of the Op-PnC and MGR, the minimum transmission for the Op-PnC reached approximately −50.0 dB, which is significantly better than the −35.0 dB achieved for the MGR. Referring to the dispersion diagram presented in Figure 1b, the minimum transmission point is between the lower and upper bounds of the bandgap. This significant reduction in transmission validates the PnC’s superior acoustic wave attenuation capability, even with just 10 lattice periods.

### 2.2. Influence of Pillar Shape on Bandgap

**Figure 2:**
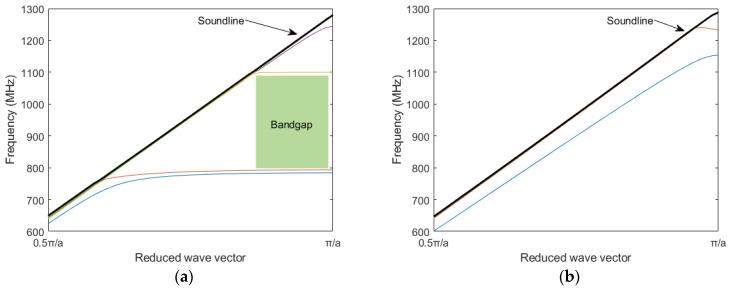
(**a**) Dispersion characteristics of Cp−PnC with *H*_pillar_ = 700 nm and *ff* = 0.3. (**b**) Dispersion characteristics of Sp−PnC with *H*_pillar_ = 700 nm and *ff* = 0.3.

Cylindrical pillar PnC (Cp-PnC) and square pillar PnC (Sp-PnC) were studied with a similar lattice constant and filling fraction to see the impact of shape of the pillar on bandgap characteristics. Figure 2a,b shows the dispersion characteristics of Cp-PnC and Sp-PnC, respectively. The results clearly show that the Cp-PnC exhibits an acoustic bandgap in the frequency range comparable to that of the Op-PnC. In contrast, the Sp-PnC lacks a bandgap in the same frequency range. This can be attributed to the higher symmetry of the Cp-PnC and Op-PnC structures compared to the Sp-PnC structure. In addition to this, even though it is not included here, the Cp-PnC shows similar acoustic bandgap dependence characteristics with the radius (*R*) and thickness of the pillar (*H*_pillar_), similar to the one shown in Figure 1c,d.

Though our study was limited to only three different shapes of pillars for the sake of easy fabrication, it is recommended to explore other pillar shapes and decide the best pillar shape for a better reflection of surface acoustic waves.

### 2.3. Influence of Pillar Material on Bandgap

In addition to the PnC shape and PnC geometric dimensional values demonstrated above, the bandgap size and bandgap center frequency ((*f*_lower_ + *f*_upper_)/2) are influenced by the material properties of the pillars, particularly their acoustic velocity. To demonstrate this, we compare four different materials commonly used for in the acoustic devices fabrication processes, aluminum (Al), copper (Cu), gold (Au) and tungsten (W), with their mechanical properties given in Table 1.

**Figure 3:**
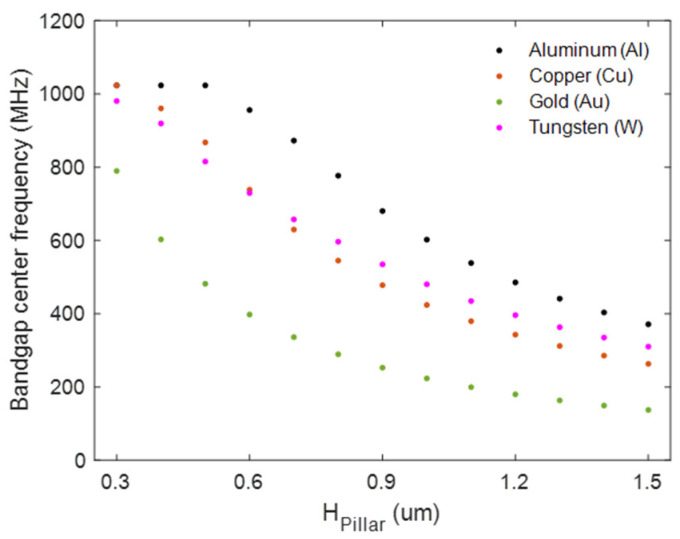
The center frequency changes as *H*_pillar_ varies from 300 nm to 1500 nm with a fixed lattice constant, *a* = 1300 nm, and filling fraction, *ff* = 0.5, across four distinct metals: aluminum (Al), copper (Cu), gold (Au) and tungsten (W).

Figure 3 illustrates the bandgap center frequency for different metals as *H*_pillar_ varies from 300 nm to 1500 nm with fixed values of *a* = 1300 nm and *ff* = 0.5. The plot clearly demonstrates that Al with the highest acoustic velocities results in higher bandgap center frequencies across the full range of *H*_pillar_, while gold (Au) with lower acoustic velocities results in lower bandgap center frequencies.

### 2.4. Application in SAW Resonators

**Figure 4:**
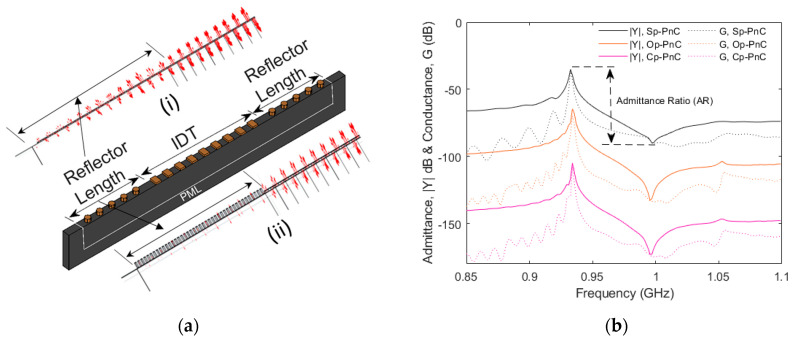

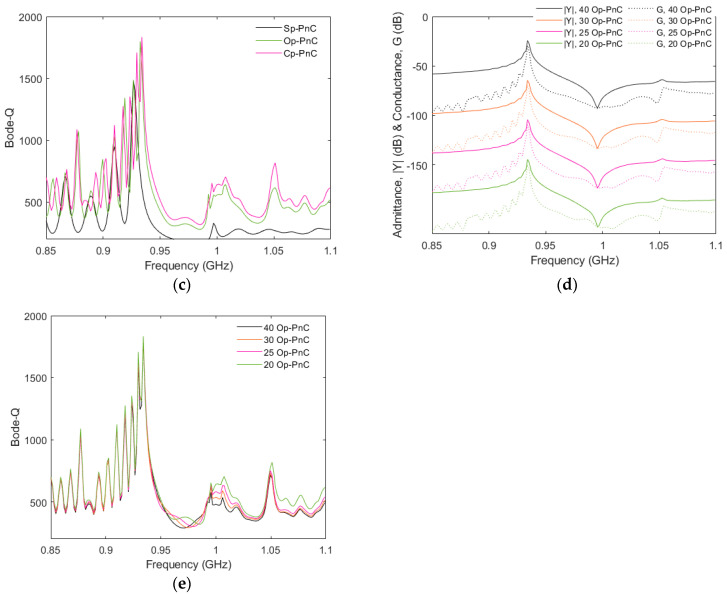
(**a**) Schematic representation of 2.5D SAW resonator model with Op−PnC reflector (size is not to scale and number of PnCs and IDT is for demonstration purposes only). (**b**) Simulated admittance (|*Y*|) and conductance (G) responses of resonators with Cp−PnC, Op−PnC and Sp−PnC reflectors. (**c**) Simulated bode-Q of resonators with Cp−PnC, Op−PnC and Sp−PnC reflectors. (**d**) Simulated admittance (|*Y*|) and conductance (G) responses of resonators with Op−PnC reflectors, with different reflector lengths. (**e**) Simulated bode-Q of resonators with Op−PnC reflectors, with different reflector lengths (40 dB shift is used among responses in (**b**,**d**) for better visibility).

To demonstrate the practical applicability and effectiveness of the pillar-based PnCs, one-port SAW resonators were designed with Op-PnC, Cp-PnC and Sp-PnC reflectors. Figure 4a displays a 2.5D schematic diagram of the SAW resonator with an Op-PnC-based reflector used for the analysis. A resonator with 101-pair interdigital transducers (IDTs) with a metallization ratio (*MR*) of 0.5, deposited on a 42° Y-X LiTaO_3_ piezoelectric substrate, is considered. Al is used for both PnC structures and IDTs. The wavelength (λ) is set at 4 µm and electrode thickness (*t*_ele_) is set at 0.07λ. PnC arrays are positioned on both sides of the IDTs to serve as reflectors. Periodic boundary conditions are applied in the y-direction, and a PML is used in the x- and z-directions to minimize reflections. The insets in Figure 4a display the stress distribution in the reflector region for resonators with standard metal reflectors (*i*) and Op-PnC reflectors (*ii*). It is evident that the resonator with an Op-PnC reflector has a significantly smaller stress field distribution compared to standard metal grating reflectors in the reflector region.

Figure 4b,c demonstrate the simulated admittance (|*Y*|) and bode-Q responses of resonators incorporating different types of PnC reflectors. The results reveal that resonators with Op-PnC and Cp-PnC reflectors achieves an admittance ratio (*AR*) of about 70 dB. But the resonator with Sp-PnC achieves a relatively smaller *AR*, specifically less than 40 dB. The bode-Q for resonators with Sp-PnC reflectors is also much smaller than that achieved for the Op-PnC and Cp-PnC.

We conducted an analysis of SAW resonators with Op-PnC-based reflectors with varying lengths to investigate the impact of the reflector length, specifically ranging from 20 Op-PnCs to 40 Op-PnCs. Figure 4d,e demonstrate the simulated admittance (|*Y*|) and bode-Q responses of resonators with Op-PnC reflectors of different lengths (40, 30, 25, and 20), respectively. The data in both figures demonstrate that the length of reflector does not have a significant impact on the performance of the resonator, particularly for the specified lengths. This may be due to the fact that the reflector length of 20 Op-PnCs is sufficient to offer adequate reflection. Thus, further increasing the reflector length does not have a significant impact on the key performance metrices.

## 3. Device Fabrication

**Figure 5:**
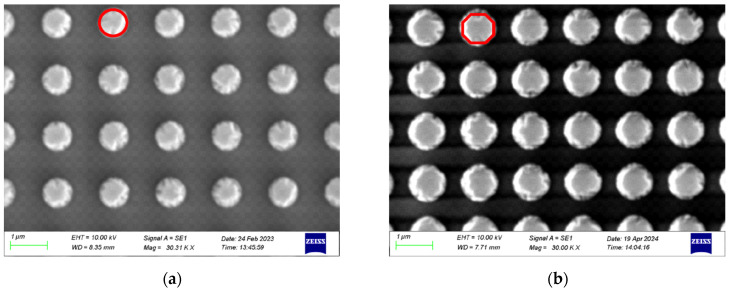

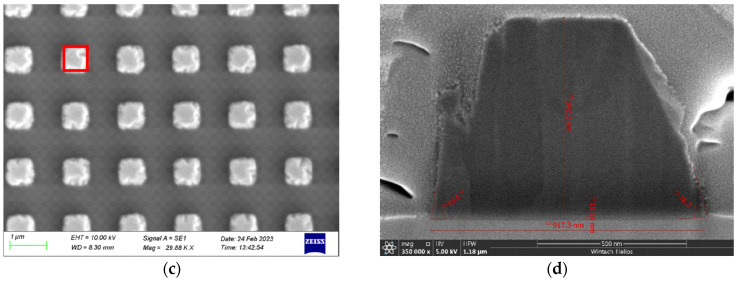
SEM images of fabricated phononic crystals. (**a**) Cp−PnC, (**b**) Op−PnC, (**c**) Sp−PnC, and (**d**) cross-sectional view of Op−PnC (the intended geometry of the pillars is highlighted in red in (**a**–**c**) for clarity).

To validate our theoretical analysis, we fabricate a series of SAW resonators and filters using lift-off and deep ultraviolet (DUV) photolithography. We use a two-mask approach as there is significant thickness difference between the interdigital transducers (IDTs) and phononic crystal (PnC) pillars; 0.07λ (280 nm) for IDTs and 850 nm for PnC pillars. For this reason, two different photoresist layers are employed: one 810 nm thick for the IDTs and another 1100 nm thick for the PnC pillars. The process begins with substrate preparation, followed by the deposition of the titanium (Ti) adhesion layer: 5 nm for the IDT and a 15 nm for the PnC. Then, subsequent material layers for the IDTs and PnC pillars were deposited with each layer patterned using its designated mask. The devices are inspected using scanning electron microscopy (SEM), with Figure 5a–c showing the SEM images of the Cp-PnC, Op-PnC, and Sp-PnC pillar arrays, respectively, and Figure 4d providing a cross-sectional view of the Op-PnC pillar structure.

## 4. Discussion

**Figure 6:**
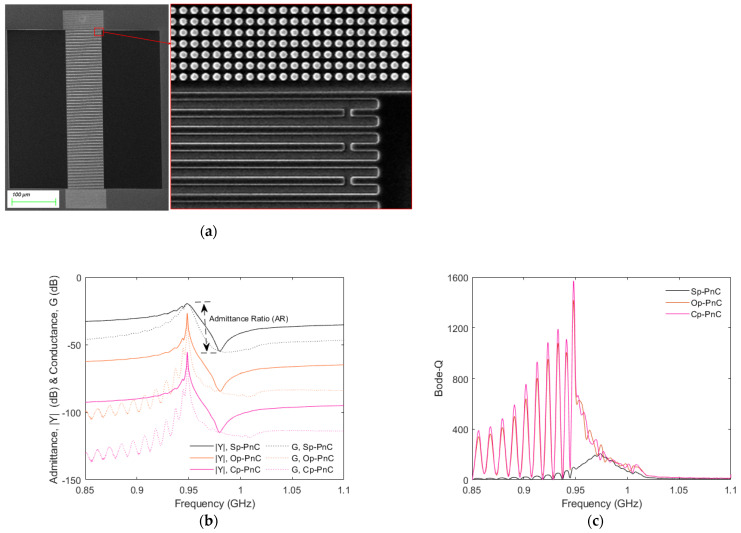
(**a**) SEM image (400× magnification) of fabricated one-port SAW resonator with Op−PnC-based reflector. (**b**) Measured admittance magnitude (|*Y*|) and conductance (G) with Cp−PnC, Op−PnC and Sp-PnC reflectors. (**c**) Measured Bode−Q plot for resonators with Cp−PnC, Op−PnC and Sp−PnC reflectors (40 dB shift is used among responses in (**b**) for better visibility).

Figure 6a shows an SEM image of a sample resonator with an Op-PnC array reflector. The zoomed-in view reflects that the PnC array is well aligned despite being patterned with a different mask from the IDTs. Figure 6b,c illustrate the measured admittance (|*Y*|) and the bode-Q plots for resonators with Cp-PnC, Op-PnC and Sp-PnC reflectors. The admittance response (|*Y*|) and bode-Q values reveal that resonators with Cp-PnC- and OP-PnC-based reflectors achieve better admittance ratios (*ARs*) of 60 dB and bode-Q with a maximum value close to 1600, which is a relatively high value for conventional SAW resonators manufactured on bulk 42° Y-X LiTaO_3_ wafers. In contrast, the resonator with Sp-PnC exhibits a significantly lower admittance ratio (*AR*) of approximately 35 dB and a maximum bode-Q of only 200, which is about one-eighth of the values achieved by the other two types of PnCs.

**Figure 7:**
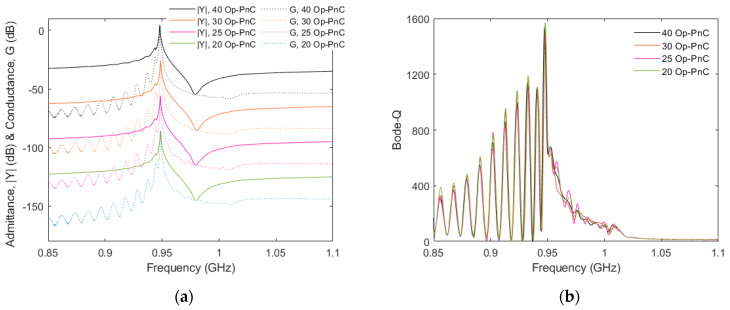
Measured characteristics of one-port SAW resonators with different Op−PnC reflector lengths (i.e., 40, 30, 25, 20 Op−PnCs). (**a**) Admittance (|Y|) and conductance (G), and (**b**) Bode−Q (40 dB shift is used among responses in (**a**) for better visibility).

Figure 7a,b show the measured admittance (|*Y*|) and bode-Q of resonators with different lengths of PnC-based reflectors, specifically, 40, 30, 25, and 20 Op-PnCs. Consistent with the simulated results shown in Figure 4d,e, the measured data also reflect that a reflector length of 20 PnC arrays provides sufficient reflection; as a result, there is no significant change in the admittance and bode-Q responses when the reflector length is increased beyond 20 PnC arrays.

**Figure 8:**
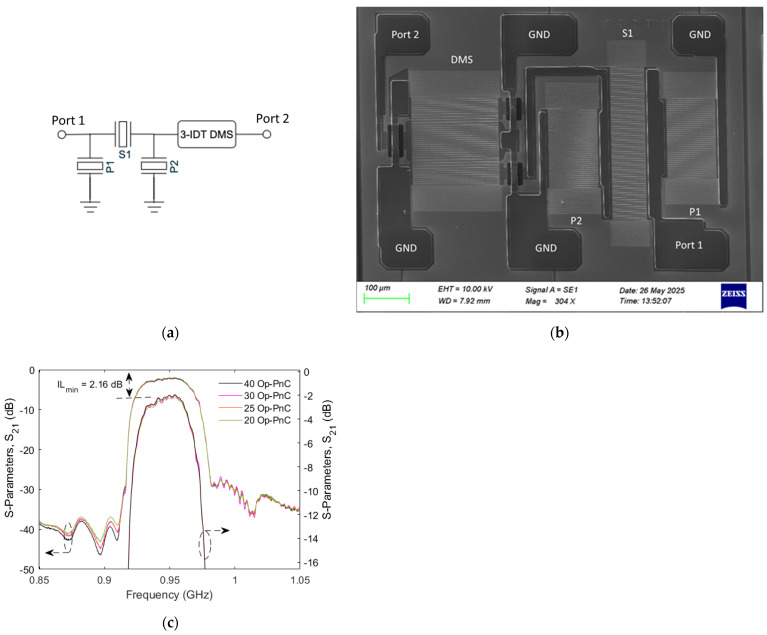
(**a**) Schematic diagram of the filter, (**b**) fabricated filter with PnC−based reflectors, and (**c**) transmission magnitude (S−Parameter, S21 (dB)) response of filters with Op−PnC reflectors of different lengths (i.e., 40, 30, 25, 20 Op−PnCs).

We implement a Band-8 RX stand-alone filter incorporating a PnC-based reflector. The filter consists of three-IDT double-mode SAW (DMS) and ladder-type synchronous resonators, including two shunt resonators (P1 and P2) and one series resonator (S1). Figure 8a,b present a schematic of the filter and SEM picture of the fabricated filter, respectively. Figure 8b shows the measured transmission (S_21_ dB) response of filters with Op-PnC reflectors with different reflector lengths, specifically with 40, 30, 25, and 20 Op-PnCs.

All the filters demonstrate promising performance, with a minimum insertion loss of less than 3 dB and an out-of-band attenuation exceeding 35 dB. However, the filter roll-off performances at both the right and left shoulders remain suboptimal and require further improvement. As such, it is evident that the overall filter performance demonstrated does not yet meet the stringent requirements of the market, but the authors think that there is a significant potential for improvement.

## 5. Conclusions

We demonstrated a nanoscale phononic crystal (PnC)-based reflector for surface acoustic wave (SAW) resonators and filters, validated through both theoretical analysis and experimental verification. Three distinct PnC pillar structures were analyzed to understand how the shape of PnC influences its dispersion characteristics. Additionally, the effects of geometric dimensions of the PnCs, specifically pillar thickness and filling fraction, on achievable acoustic bandgap were explored. To this end, various pillar thicknesses and filling fractions (i.e., different pillar radii, *R*) are systematically analyzed. Furthermore, the study examines the impact of reflector length on the performance of PnC-based reflectors. A range of reflector lengths, from 20 to 40 PnC arrays, is considered both in simulations and fabricated devices. The applicability of PnC-based reflectors is demonstrated by incorporating them in SAW resonators and filters. Specifically, the resonators with the proposed PnC-based reflector design, using arrays of Cp-PnC and Op-PnC reflectors, achieved a maximum bode-Q of about 1600, while devices with the Sp-PnC reflector achieved a bode-Q of about 200 only. Furthermore, we demonstrated filters based on OP-PnC array reflector with different lengths and achieved a minimum insertion loss of less than 3 dB regardless of the reflector size. The authors believe that with an improvement of the fabrication process, PnC-based reflectors could manage to be able to achieve the performance requirements of the state of the art and help to greatly reduce the area occupied by the reflector, resulting in device miniaturization.

## Figures and Tables

**Table 1:** Summary of mechanical properties of different metals [39].

Metal	Density (ρ) (g/cm^3^)	Acoustic Velocity (*V*) (m/s)			Acoustic Impedance (*Z*) (MRayl) ^1^
		Longitudinal	Shear	Extensional	
Aluminum (Al)	2.7	6420	3040	5000	~17.1
Copper (Cu)	8.96	4760	2325	3810	~42.1
Gold (Au)	19.32	3240	1200	2030	~62.2
Tungsten (W)	19.25	5410	2640	4320	~100.5

^1^ Acoustic impedance (*Z*) = Density (ρ) × Acoustic Velocity (*V*).

## Data Availability

The data presented in this article are available on a reasonable request from the corresponding authors.
